# Advances in Oral Drug Delivery

**DOI:** 10.3389/fphar.2021.618411

**Published:** 2021-02-19

**Authors:** Mohammed S. Alqahtani, Mohsin Kazi, Mohammad A. Alsenaidy, Muhammad Z. Ahmad

**Affiliations:** ^1^ Department of Pharmaceutics, College of Pharmacy, King Saud University, Riyadh, Saudi Arabia; ^2^ Nanobiotechnology Unit, Department of Pharmaceutics, College of Pharmacy, King Saud University, Riyadh, Saudi Arabia; ^3^ Department of Pharmacognosy, College of Pharmacy, King Saud University, Riyadh, Saudi Arabia

**Keywords:** nanoparticles, lipophilic, stomach, oral drug delivery, biodegradable, solubility

## Abstract

The oral route is the most common route for drug administration. It is the most preferred route, due to its advantages, such as non-invasiveness, patient compliance and convenience of drug administration. Various factors govern oral drug absorption including drug solubility, mucosal permeability, and stability in the gastrointestinal tract environment. Attempts to overcome these factors have focused on understanding the physicochemical, biochemical, metabolic and biological barriers which limit the overall drug bioavailability. Different pharmaceutical technologies and drug delivery systems including nanocarriers, micelles, cyclodextrins and lipid-based carriers have been explored to enhance oral drug absorption. To this end, this review will discuss the physiological, and pharmaceutical barriers influencing drug bioavailability for the oral route of administration, as well as the conventional and novel drug delivery strategies. The challenges and development aspects of pediatric formulations will also be addressed.

## Oral Drug Delivery

Oral medication is the most common form of drug administration because of advantages such as convenience of drug administration via the oral route, patient preference, cost-effectiveness, and ease of large-scale manufacturing of oral dosage forms. Around 60% of established small-molecule drug products available commercially are administered via the oral route. Current estimates indicate that oral formulations represent about 90% of the global market share of all pharmaceutical formulations intended for human use. Around 84% of the best-selling pharmaceutical products are orally administered and are currently valued at $35 billion, with an annual growth rate of 10% (Prasad et al., 2017).

**FIGURE 1:**
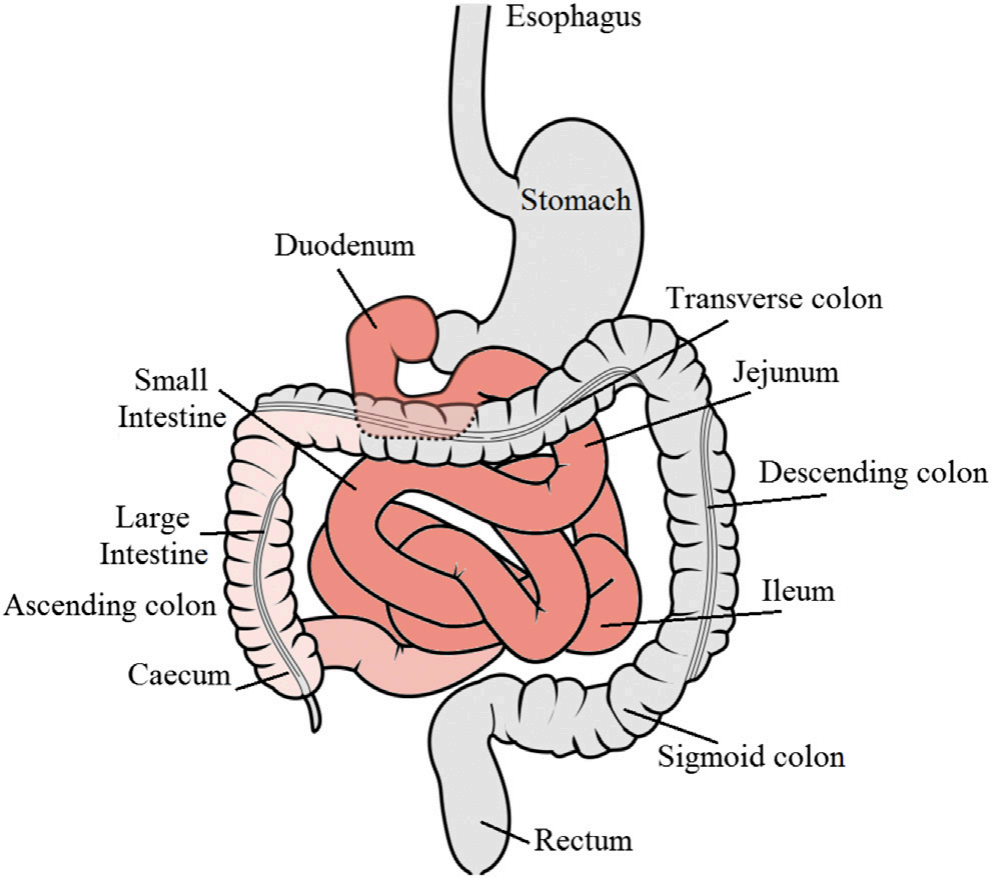
Schematic diagram of the gastrointestinal tract showing the major regions for drug absorption denoted in red color. GI tract diagram by Olek Remesz (https://commons.wikimedia.org/wiki/File:GISystem.svg), under a Creative Commons license.

The compliance of patients to oral formulations is generally higher than that to other parenteral routes such as intravenous, subcutaneous, and intramuscular injections, as well as to inhalation for asthma medications (Ingersoll and Cohen, 2008). Furthermore, orally administered drugs can be targeted to particular regions within the gastrointestinal (GI) tract for localized treatment of pathological conditions such as stomach and colorectal cancers, infections, inflammations, bowel diseases, gastro-duodenal ulcers, and gastroesophageal reflux disorders (Figure 1).

Despite these advantages, the development of oral formulations presents several challenges, which are mainly attributed to the physicochemical properties of drugs, including poor water solubility and membrane permeability. In addition, the absorption of drugs can be limited by their poor chemical and biological stability, as well as by physiological barriers, including pH, efflux transporters, and metabolic enzymes. Further, some drugs can cause local irritation and nausea (Rubbens et al., 2018). Over the last four decades, numerous studies have focused on understanding the mechanism of drug absorption and transport, intestinal transit, microenvironment of the GI tract, and drug stability in the GI fluids (Daugherty and Mrsny, 1999; Reix et al., 2012). Thus, the elaboration of oral drug delivery systems necessitates a thorough understanding of the physicochemical properties, GI permeability, biological barriers, pharmacokinetics, and pharmacodynamics of drugs.

### Biological Barriers

Most orally administered medications are primarily absorbed by the duodenum and jejunum in the upper parts of the GI tract. The drug absorption ability of the stomach is less than that of the intestine because of the smaller surface area and thicker mucus layer (thickness, 1.5 mm) of the former (Table 1) (Mudie et al., 2010). The epithelial lining of the intestines is one of the major barriers to drug absorption in the GI tract. Epithelial cells are arranged in a single-column layer, and the building blocks, which are intercalated with enterocytes and joined by zonula occludens or tight junctions, are present at their apical surface. The tight junctions are mainly accountable for the passage of hydrophilic molecules via paracellular route. The epithelium on the apical surface projects with the lamina propria to form villi that contain microvilli. About 3,000–7,000 microvilli per cell in the small intestine provide a large surface area for drug interaction and absorption (Zhuu et al., 2017). Although the structures of the microvilli remarkably increase the surface area for absorption in the small intestine, they additionally provide an enzymatic barrier since their brush border is concentrated with digestive enzymes (Zupančič and Bernkop-Schnürch, 2017).

**TABLE 1:** Physiological features of the human gastrointestinal tract.

GI tract region	Approx. length (m)	Approx. surface area (m^2^)	pH	Epithelial type	Approx. residence time	Major enzymatic activities
Oral cavity	–	0.01	6.5	Stratified Squamous	–	Polysaccharidase
Esophagus	0.2–0.25	0.02	–	Stratified Squamous	4–8 s	–
Stomach	0.25	3.5	1.0–3.0	Secretory Columnar	1–3 h	Proteases, lipases
Duodenum	0.35	1.9	4.0–5.5	Simple columnar	30–40 min	Polysaccharidase, oligosaccharidases, proteases, peptidases, lipases
Jejunum	2.8	184	5.5–7.0	Simple columnar	1.5–2.0 h	Oligosaccharidases, peptidases, lipases
Ileum	4.2	276	7.0–7.5	Simple columnar	5–7 h	
Colon	1.5	1.3	7.5–8.0	Columnar dominated	16–35 h	Broad spectrum of bacterial enzymes
Rectum	0.12	–	7.0	Columnar dominated	–	

Absorption of drugs from the lumen of the GI tract requires their passage through multiple layers including gastric juice, pericellular matrix, and mucous rich layer, to reach the epithelium, mucosa, and blood or lymph capillary walls. Therefore, bioadhesive drug delivery systems often exhibit improved performance compared to matrix tablets. Bioadhesive microspheres can diffuse into the mucous gel layer because of the small size of the nanocarriers and show a prolonged gastric residence time (Vasir et al., 2003). The maintenance of a bioadhesive system in the stomach for an extended time facilitates the treatment of both local diseases as well as prolonged drug absorption for systemic delivery.

Another factor that influences drug absorption is the pH of the GI fluid. In the fasting state, the stomach pH varies, and the median basal pH for adult males is 2.18 ± 0.18 (Goldschmiedt et al., 1991). Thus, drugs with poor stability under acidic pH need to be protected in the stomach. Pepsin, which plays an important role in digesting most of the ingested proteins, is active at acidic pH, but is promptly inactivated above pH 4 (Rouge et al., 1996). Thus, a sufficient amount of pH-increasing buffer that raises the local stomach pH to values above 4 can deactivate pepsin. Enteric polymer coatings such as acetate phthalate and methacrylate-based polymers can be used to protect drugs that are unstable under acidic pH conditions of the stomach (Chen et al., 2000). Unlike the stomach, the duodenum is a highly permeable region of the intestine with neutral pH (Zhu et al., 2017).

The GI transit time is also important for developing an oral dosage form. In humans, the transit time of drug dosage forms through the small intestine is constant with a universally accepted value of 3 h and is independent of the physical characteristics of the dosage forms, such as density and size, as well as of food (Dressman and Reppas, 2016). However, the gastric transit time is known to vary and so does the drug bioavailability. This variability might eventually lead to unpredictable levels of drug plasma and can severely limit the clinical efficacy. Gastrointestinal movements are of two types: propulsive and mixing; they are mainly affected by the fed or fasted state as well as the sleep cycle. The peristalsis motilities primarily determine the passage rate and thus, the residence time of a drug after oral administration (Rouge et al., 1996). In humans, the intestinal content has been shown to pass through the intestinal tract at a rate of 3 cm/min (Said and Mohammed, 2006). The passage rate is higher in the upper parts of the intestinal tract and declines toward the ileum. A drug capsule requires 3–4 h to pass through the entire small intestine. However, the transit time is considerably greater in the large intestine and depends on the volume of fiber in the intake. In healthy humans, the route time through the large intestine is estimated to be around 2 to 4 days (Read et al., 1980). The residence time in the intestine also imitates the absorption of drugs that are poorly soluble or that dissolve slowly in the intestinal fluids, as well as of the pharmaceutical formulations that sustain the release of the drug. Furthermore, the transit or residence time is essential for small drug molecules that are absorbed by transport carriers, as these drugs are favorably absorbed in the location with the highest carrier density (Dressman and Reppas, 2016). For instance, vitamin B2 is absorbed mostly in the proximal small intestine via sodium-dependent, carrier-mediated transport (Said and Mohammed, 2006). Hence, influences that effect intestinal motility can impact the bioavailability of vitamin B2. Thus, the extent of drug absorption after oral administration is directly affected by the GI residence time (Sakr, 1999).

Food can influence the absorption of drugs: it can decrease, increase, delay, or accelerate drug absorption (Custodio et al., 2008). Food affects the GI functions such as gastric emptying, intestinal transit time, bile acid secretion, stomach pH change, and liver blood flow increase. Further, it can alter the physiochemical characteristics of drugs, such as solubility, intestinal permeability, size, and dissolution profile. In general, hydrophobic drugs or drugs with solubility that is pH-dependent are mainly manipulated by the co-administered food (Cheng and Wong, 2020). It is known that, high-fat meals increase the concentrations of the pancreozymin (cholecystokinin), which stimulates gallbladder secretion of bile within the GI tract. This leads to the formation of solubilizing micellar carriers, which can assist in the solubilization of drugs and their absorption from the lumen of the GI tract (Shneider, 2001). Certain fruit juices are known to either affect the transport and metabolism of drugs or enhance the extent of drug absorption (Ameer and Weintraub, 1997). The effects of grapefruit juice have been extensively studied, although studies on other juices, including orange, tangerine, lime, and apple, have been performed. From the perception of drug metabolism, the inhibition of cytochrome P450 3A4 (CYP3A4) enzyme has been associated with the drug transport and metabolism inhibition effect of these juices. Further, some components such as flavonoids and furanocoumarins in some of these juices inhibit P-glycoprotein (P-gp) and organic anion transporters (Guo et al., 2000).

As drugs travel throughout the GI tract, they have the potential to cross the mucous membranes of the GI organs including the mouth, esophagus, stomach, duodenum, jejunum, ileum, and colon. If they are not able to cross the membranes by the time they reach the colon, they end up eliminated in the feces and will not be completely absorbed by the intestine. Following oral administration, the dissolution of a drug starts when it comes in contact with the GI fluids, followed by the penetration of the aqueous medium into the dosage form, which generally contributes in the disintegration of the solid dosage into fine particles. The next step includes the mixing of the drug molecule into the dissolution medium. The dissolution process has been studied by Wagner (1970). Drug molecules in solution can cross the mucosal membrane of the GI tract via several mechanisms that include passive diffusion or active drug transport. Passive diffusion involves two distinguished routes: the paracellular route, in which drugs diffuse through the small pores at the tight junctions between the mucosal enterocytes; the transcellular route, which involves lipophilic drug diffusion across the cell membrane phospholipid of intestinal enterocytes. Active drug transport is facilitated by cell membrane transporters and is divided into active influx of drug and efflux pump. The significance of each mechanism is determined by the physico-chemical characteristics of drug molecules and their affinity for different transporter proteins (Mannhold et al., 2009; Dahlgren and Lennernäs, 2019).

The transcellular route is the main pathway of absorption for the smallest drug molecules. Overall, the absorption via the transcellular route is basically due to diffusion down a concentration gradient, and the rate of absorption is primarily determined by the rate of drug transport across the intestinal membrane, which is dictated by the physico-chemical properties of a drug. However, in the paracellular pathway, nonionized lipophilic drugs with molecular weight of more than 300 g/mol are absorbed via the transcellular pathway. In addition, the hydrogen-bonding capability of the drugs dictated by the number of hydrogen bond donors and acceptors should be less than 10 and 5, respectively (Lipinski, 2000; Avdeef, 2001).

In paracellular transport, drug molecules are absorbed by diffusion and convective volume flow through aqueous intercellular spaces (Hayashi et al., 1997). In common, drugs that are absorbed via this route are small hydrophilic molecules with molecular weight less than 200 g/mol. Moreover, since the junctional complex of the intestinal epithelium has an overall negative charge, cationic molecules pass through more freely (DiMarco et al., 2017). Nevertheless, absorption via this pathway is mostly low as the tight junctions between cells with a pore diameter of 4–8 Å limit free *trans*-epithelial passage of most drug molecules across the intestinal membrane. In addition, the paracellular transport represents around 0.1–0.01% of the total surface area of the intestinal membrane and becomes less accessible from the jejunum toward the colon, thereby providing only a limited window for drug absorption (Sugano et al., 2002).

Unlike passive diffusion of drug, carrier-mediated transport requires the interaction of drug molecules with a protein carrier, usually in the apical membrane of the enterocyte cells. Several transporters belonging to the adenosine triphosphate (ATP) binding cassette transporters (ABC transporters) superfamily and solute carrier (SLC) transporters are expressed in the apical and basolateral membranes of the GI tract for the influx or efflux of endogenous substances and xenobiotics. The absorption via this pathway is an energy-consuming process requiring ATP hydrolysis and can occur against a concentration gradient, that is, from a region of lower drug concentration to that of higher concentration. Although diverse transporters are exhibited in the enterocytes, only a limited number of transporters are known to play an important role in the intestinal absorption of drugs (Müller et al., 2017). For instance, ABC transporters superfamily utilizes ATP to initiate the transport and are called primary active transporters. For example, methotrexate, a folic acid antagonist, was found to be absorbed via the ABC-dependent, proton-coupled folate transporter/heme carrier protein 1 in the proximal small intestine (Yokooji et al., 2009). Conversely, SLC transporters mainly use the ion gradients (hydrogen, calcium, and sodium ion gradients) created within the cellular membrane by primary active carriers (Na^+^/K^+^-ATPase and Na^+^/H^+^-ATPase) (Tsuji and Tamai, 1996). ABC transporters distributed and expressed in the intestinal epithelium include permeability glycoprotein (P-gp), breast cancer resistance protein (BCRP), and multidrug resistance (MDR) proteins. P-glycoprotein 1, BCRP, MDRP2, and MDRP4 are expressed on the apical side of the membrane, whereas specific MDRPs are localized on the basolateral membrane of the absorptive epithelial cells. These membrane transporters functionally minimize the cellular levels of their substrates by decreasing uptake and enabling the efflux pump. By contrast, the facilitated drug absorption involves a protein carrier but does not involve energy. The drug concentration gradient, as in passive diffusion, is the main driving energy for this absorption pathway. The common examples of facilitated absorption are the intestinal uptake of glucose, folate, and vitamin B12 (Steffansen et al., 2004).

### Physicochemical Barriers

The absorption of drugs in the GI tract require their release from the dosage form; the released drug dose need to be in a solution form or should have the ability to dissolve in the GI fluid. Further, the dissolved drug must be permeable through the intestinal membrane. Therefore, the aqueous solubility and intestinal epithelial membrane permeability of drugs are the critical determinants of GI absorption; these criteria form the basis for the classification of drugs into four categories by the Biopharmaceutical Classification System (BCS; Table 2) (Amidon et al., 1995). In the BCS, the solubility criteria are based on the highest dose strength that can dissolve in a glass of water (250 ml; volume) or less of aqueous media over a pH range of 2–7.5. (Yu et al., 2002). Permeability is often referred to as the diffusion across the apical membrane of enterocytes into the cytosol and depends on drug properties such as polarity, charge, and lipophilicity (Lennernäs, 2007). A drug is known to be highly permeable if the percentage of absorption is ≥90% of the administered dose. BCS Class I drugs have high solubility and permeability and are good candidates for oral delivery. Conversely, other BCS classes are challenging candidates for oral delivery owing to their low solubility (BCS Class II), low permeability (BCS Class III), or both (BCS Class IV). The oral absorption ability of BCS Class II drugs can be improved by increasing their dissolution rate.

**TABLE 2:** The biopharmaceutics classification system.

Class I	Class II
High solubility	Low solubility
High permeability	High permeability
Class III	Class IV
High solubility	Low solubility
Low permeability	Low permeability

In addition to solubility and permeability, drug metabolism can also influence their oral bioavailability. Hence, Wu and Benet proposed the Biopharmaceutics Drug Disposition Classification System (BDDCS) (Wu and Benet, 2005). The BDDCS provides understandings into the effects of diet on drug absorption and information on the interplay between drug absorption, elimination, and transport. According to the BDDCS, drug permeability is influenced by the major route of elimination. Class 1 BDDCS drugs, which have high solubility and are considerably metabolized, are not expected to display significant transporter drug interactions. Thus, high-fat meals should have no significant effect on the extent of the bioavailability of such drugs. However, high-fat meals delay stomach emptying and reduce absorption and thus increase the *T*_max_ (Custodio et al., 2008). Class 2 BDDCS drugs, which are poorly soluble and highly metabolized, might be subjected to significant transporter effects, mainly efflux transporter effects, due to their insolubility. Therefore, high-fat diets might increase their bioavailability owing to the inhibition of efflux pump such as P-gp transporters in the intestine. Dosage form changes that significantly increase the solubility of BDDCS class 2 drugs might decrease or eliminate the effect of high-fat meals and mostly minimize other drug transporter interactions. Class 3 BDDCS drugs are known to be more vulnerable to the effect of uptake transporters owing to their low permeability. Fatty diets can reduce the bioavailability of these drugs owing to the inhibition of intestinal uptake transporters. However, if a drug is a substrate for transporters (influx or efflux), the main effect will depend on the degree of transporter inhibition, as well as on the substrate’s relative affinity for the transporters. This can result in either an unpredicted increase in the drug bioavailability or no effect (Dressman and Reppas, 2016). For class 4 BDDCS drugs, predicting the effect of a high-fat meal on drug absorption is difficult, as a combination of interactions of both class 2 and 3 compounds is possible. Conversely, when fatty diet effects appear, they are mostly exhibited by an increase in the drug bioavailability, resulting from the combination of enhanced solubilization of a drug in the GI, as well as the inhibition of efflux transporters.

### Metabolic and Biochemical Barriers

Intestinal metabolism is normally triggered by digestive enzymes secreted by the pancreas, such as lipases; amylase; and peptidases, including chymotrypsin and trypsin, as well as those that are originated from the intestinal flora of the colon found mainly within the lower part of the GI tract. In addition, the first-pass metabolism, which includes intracellular and brush-border metabolism, occurs on the enterocyte surface by enzymes present within the membrane of the brush border. Brush-border metabolism occurs mainly in the small intestine. Isomaltase, alkaline phosphatase, sucrose, and other peptidases contribute to the brush border metabolism (Barthe et al., 1999). First-pass metabolism might limit oral absorption.

Intracellular metabolism occurs in the enterocytes and mainly involves phase-I metabolizing enzymes, including cytochrome P450 enzymes such as CYP3A4; several phase-II conjugating enzymes associated with reactions such as sulfation and glucuronidation; and other enzymes such as esterases (Gibson and Skett, 2001). Although the intestinal epithelium is a site for pre-absorptive metabolism, it can act as a major site for the delivery of ester-type pro-drugs such as aspirin (Thummel et al., 1997). In addition to the intestinal epithelium, hepatic first-pass metabolism represents the major metabolic barrier.

Membrane transporters can be categorized into two types: uptake and efflux transporters; they facilitate the transport of drugs and endogenous compounds out or into the cells. Thus, membrane transporters are important determinants for oral drug absorption, disposition, and bioavailability (Shugarts and Benet, 2009). The main uptake transporters that enable xenobiotic transport of drugs into the cells belong to the solute carrier (SLC) superfamily, whereas the efflux transporters belong to the ABC superfamily (Giacomini et al., 2010). In the liver and intestine, efflux transporters, including bile salt export pump (BSEP), Pgp, MRP1-6, and BCRP, are highly expressed. Most of these membrane transporters utilize ATP to pump substrates against a concentration gradient. In the small intestine and largely in the colon, P-gp is mainly located in the brush border surface of enterocytes where it acts as a defense barrier against exogenous compounds. Furthermore, CYP3A4 is co-localized with P-gp in mature enterocytes and has overlapping substrate specificity (Watkins, 1997). Thus, most of substrate drugs might be metabolized by pumping them out of the enterocytes into the lumen via P-gp before they can be reabsorbed again into the cells, thereby prolonging their exposure to CYP3A4 (Watkins, 1997). This mechanism limits the bioavailability of many drugs (Gibson and Skett, 2001). Moreover, it can lead to drug-drug interactions, especially when drugs are made to inhibit P-gp or CYP3A4 (Thummel, 2007). The main factors that affect drug absorption after oral administration are summarized in Table 3.

**TABLE 3:** Factors that affect drug absorption from the gastrointestinal tract.

Physiological factors	Physicochemical factors	Formulation factors	Miscellaneous
I. Physiology of GIT a. pH of various segments b. Esophageal transit time c. Esophageal motility d. Presence or absence of food II. Mode of transport across the GI tract a. Passive diffusion b. Active transport III. Metabolism	i. Drug stability in the GI fluid ii. Ionization constant iii. Lipophilicity of the drug iv. Drug solubility v. Crystal properties vi. Dissolution rate vii. Salt form viii. Protein binding ix. Complex formation x. Adsorption	i. Solutions ii. Suspensions iii. Capsules iv. Tablets v. Coated tablets	i. Age ii. Gender iii. Smoking and Alcohol abuse iv. Other drug use

### Strategies to Improve Oral Drug Delivery

Development of oral formulations for drugs with poor aqueous solubility requires the understanding of barriers. Drug solubility is a key element of the low oral bioavailability of hydrophobic drugs (Boyd et al., 2019). Other factors related to low bioavailability of hydrophobic drugs are food effect, gastric irritation, slow onset of action, lack of dose proportionality, and high intra- and inter-subject variability (Singh and Kim, 2002). Therefore, many approaches are utilized to improve the aqueous solubility of drugs (Table 4). Formulation considerations such as surfactant selection, particle size reduction, and salt selection need to be carefully screened to develop formulations of poorly soluble drugs. Traditionally, a combination of surfactants has been utilized for improving the oral absorption of drugs (Wong et al., 2006). Surfactants contain a hydrophilic head and hydrophobic tail, in which both the hydrophilic and lipophilic groups help the drug molecules in localizing at the interface, thereby diminishing the interfacial tension. Surfactants improve the bioavailability of drugs via several mechanisms, which include enhancing the solubility and permeability of drugs by momentarily opening tight intracellular junctions. However, the use of surfactants at higher concentrations can become a safety concern and requires careful consideration (Lawrence, 1994). Other techniques such as micro/nanonization can also improve the bioavailability of drugs to a remarkable extent (De Villiers et al., 2008; Liu, 2018). In these techniques, the particle size of pharmaceuticals is reduced considerably, which in turn increases their surface area and subsequently the dissolution rate. A brief summary of the formulation approaches for various BCS class drugs is shown in Table 5.

**TABLE 4:** Different strategies to enhance the aqueous solubility of drugs.

Type		Advantages	Limitations	References
Crystal engineering	Metastable polymorphs	Minimal amounts of surfactants and polymers are required for stabilization. High drug loading and high energy systems that are beneficial in drug dissolution	Challenges in drug/polymer miscibility, excipients compatibility for a chosen drug. Physical instability upon storage	(Blagden et al., 2007; Varshosaz et al., 2018)
	Co-crystal formation			
Chemical modification	Pro-drug formation	Improved drug solubility, lipophilicity, transporter-mediated absorption. The potential to achieve site-specific delivery	Limitations in prodrug screening and development. Associated with a higher possibility for the formation of degradation by-products and lack of chemical stability. Disruption of solid-state crystallinity and polymorphism	(Mueller, 2009; Sanches and Ferreira, 2019)
	Salt formation	The most commonly applied technique to increase solubility and the preferred approach for the development of liquid formulations. Enhanced the dissolution rate by increasing the apparent intrinsic solubility of the drug. Ease of synthesis and low cost of raw material	Restricted to weakly acidic or weakly basic drugs and is not suitable for neutral drug compounds. Conversion of the drug salt back into its respective free acid or base forms in the GI fluid after oral administration. Limitations in salt screening and the selection of optimal salt forms	(Serajuddin, 2007; Vioglio et al., 2017)
Particle size reduction	Micronization and nanosized drugs, e.g., NanoCrystal, DissoCubes	Easy to scale up and time efficient. Reduced drug degradation because the drug is in the crystallin solid-state. Feasibility of formulating a drug under different pharmaceutical dosage forms	Physicochemical-related stability issues such as aggregation or a change in the solid state of the drug. The excess use of excipients as stabilizers which may change the drug bioavailability and pharmacological activity. Bulking care is essential particularly during handling and transport	(Miiller et al., 2002; Kesisoglou et al., 2007; Williams et al., 2013)
Amorphization	Solid dispersion	Provided extra stability and protection of the drug during formulation. Enhanced solubility and dissolution rate compared with traditional crystal habit modification; it also retarded agglomeration/crystallization of drug molecules due to its molecular level dispersion and steric hindrance interactions within the polymeric matrices	The high-energy amorphous drug tends to convert and recrystallize to a low energy crystalline form. The miscibility between the selected drug and polymeric matrices is required. Limited stability is a known drawback	(Leuner and Dressman, 2000; Savjani et al., 2012; Baghel et al., 2016)
Solvent composition	pH adjustment	The simple and powerful strategy for solubility adjustment of ionizable drugs. The drug candidate is ionized to a degree that allows complete solvation of the target drug dose. This approach applies equally to drug salts or the corresponding free acid or free base drugs	The long-term effect on the drug stability. The distortion of physiological pH. The precipitation tendencies and incompatibility upon dilution	(Strickley, 2004; Jouyban, 2008; Vemula et al., 2010)
	Co-solvent	Provided the optimum solubility for nonpolar drugs by reducing solvent polarity. The presence of a cosolvent can provide additional solubilization for drug solutions where pH manipulation is insufficient	The use of co-solvents is limited to relatively few solvents. The risk of precipitation upon dilution. It may alter the pH and strength of the buffers that are contained in a drug formulation	
Drug carrier systems	Micelles	Its hydrophobic core acts as a reservoir for lipophilic drugs. Ease of chemical modification and can be stimuli-responsive	Disintegration of micelles due to their dilution after oral administration, *in vivo* instability below the critical micelle concentration. Low drug loading	(Yano et al., 2010; Keskin and Tezcaner, 2017; Zhang et al., 2017)
	Nanoparticles	Increased solubility of lipophilic drugs, enhanced drugs stability, sustained drug delivery, shielding of the drug cargo from enzymatic activity, prolonged retention in the gastrointestinal tract, improved mucoadhesiveness, overcoming multidrug resistance, the potential for targeting specific cells and uptake via M cells	Challenges in biocompatibility and safety of polymeric carriers. Toxicity as a result from high tissue accumulation of non-biodegradable NPs. Difficulties in optimizing the process parameters and to scale up the production into a pharmaceutical product	(Merisko-Liversidge et al., 2003; Hu et al., 2004; Merisko-Liversidge and Liversidge, 2008)
	Cyclodextrins	Generally recognized as safe (GRAS) excipient. Suitable for the generation of supersaturated drug solutions. Enhance both the physical and chemical stability of drugs and their shelf-life	The requirement for a large amount of cyclodextrin compared to the drug to solubilize the drug. The weak binding and dissociation of complexes upon dilution in the GIT. The intact drug/CD complexes are unable to permeate the lipophilic epithelium membranes which may result in low bioavailability especially for BCS class III drugs	(Hirayama and Uekama, 1999; Brewster and Loftsson, 2007; Kim et al., 2020)
	Lipid-based formulations (SLN, liposomes, SEDDS)	Non-immunogenic, biocompatible, can stimulate the secretion of bile salts, phospholipids and cholesterol, which form vesicles and micelles that then facilitate drug absorption, scalable and easily manufacturable	Poor stability and short shelf life	(Pouton, 2000; Ahn and Park, 2016; Menzel et al., 2018)

**TABLE 5:** Formulation approaches for various BCS class drugs.

Solubility	Permeability	BCS class	% of marketed drugs	Formulation approaches
High	High	I	35	Conventional capsule or tablet
Low	High	II	30	Nanotechnologies, micronization, self-emulsifying and microemulsifying systems, solid dispersions, surfactant-based formulations, complexation with β-cyclodextrins, and adsorption onto hydrophilic inert carriers or ion-exchange resins
High	Low	III	25	Absorption and permeability enhancers, lipid-based formulations, and ion-pairing approach
Low	Low	IV	10	Combination of approaches for classes II and III

### Salt Formation

Salt formation is the common conventional method for enhancing the oral absorption of weakly acidic and basic drugs (Serajuddin, 2007). In general, salts of weakly acidic and basic drugs have higher solubility than their corresponding pure forms. Among the salt forms approved by the Food and Drug Administration (FDA), hydrochloride and methanesulfonate (mesylate) are the most common ions for basic drugs, whereas sodium and calcium are the most common ions used for acidic drugs (Lam et al., 2010). The pH solubility profile can be used to increase the aqueous solubility of a drug by adjusting the pH. Furthermore, the capability of a salt to alter the overall medium pH is especially important because the micro-environmental conditions in the diffusion layer have been shown to represent a critical role in enhancing the dissolution rate of drug molecules (Yang et al., 2014). A basic drug with a higher pKa, maximum intrinsic solubility, and lower salt solubility has been shown to favor salt formation under increased pH; in contrast, for an acidic compound, lower pKa and increased intrinsic solubility yield a lower pH, thereby increasing the possibility for salt formation. Nevertheless, an error and trial process is required to identify and select the most suitable salt form for drugs.

### Chemical Modification

A prodrug is a chemical derivative of a main drug; it needs to undergo enzymatic biotransformation in the body to convert to an active drug. The prodrug approach is a common chemical modification to improve drug properties, including aqueous solubility, lipophilicity, stability, mucosal membrane permeability, and therapeutic index. The most common prodrug types include ester, amide, carbonate, carbamate, azo, glucuronidic, and glycosidic bonds. In addition, polar moieties such as polyethylene glycol (PEG) are commonly included in drug molecules (Greenwald et al., 2000; Basit et al., 2001). Paclitaxel is BCS class IV drug with insolubility and poor permeability; is absorption following oral administration was increased by PEGylation. The improvement of the oral absorption of a PEGylated prodrug is partially attributed to the bypass of P-gp efflux pump and cytochromes P450 metabolism (Choi and Jo, 2004; Hussain et al., 2019). In addition, the inhibitory activity of efflux pump by several conjugates such as PEG-based detergents have been reported (Veronese and Pasut, 2005). Among these detergents, polysorbate (Tweens) and tocopheryl poly(ethylene glycol) succinate 1,000 (TPEGS 1000) are the most commonly used in oral drug delivery. The prodrugs should be inert, nontoxic, and metabolizable. The prodrug design can improve the oral bioavailability of drugs by enhancing their water solubility and gastrointestinal permeability and overcoming first-pass metabolism. Prodrugs can improve the carrier-mediated absorption of charged or polar drugs with negligible passive absorption (Shah et al., 2020). Further, they can target specific bioactivation mechanisms or colon bacterial microflora to achieve site-specific drug delivery (Schacht et al., 1996). Approximately 7% of the marketed drugs are estimated to be prodrugs (Rautio et al., 2008). Lipophilic esters are the most commonly used for oral prodrugs; they can enhance drug absorption by improving membrane permeability and absorption via the lymphatic route (Charman and Porter, 1996). Some representative examples of oral prodrugs are listed in Table 6.

**TABLE 6:** Representative examples of prodrugs used for oral drug delivery.

Prodrug type	Oral application	Commercial examples
Esters	Enhancing aqueous solubility	Etoposide phosphate (Vepesid^®^)
Oxides		Sulindac (Clinoril^®^)
Esters	Improving lipophilicity and intestinal permeability	Enalapril maleate (Vasotec^®^), Ramipril (Altace^®^), olmesartan medoxomil (Benicar^®^)
Ester salts	Carrier-mediated absorption	Valacyclovir (Valtrex^®^)
Amides		Midodrine (Amatine^®^)
Carbamates		Gabapentin enacarbil (Horizant^®^)
Azo prodrugs	Colon-specific targeting	Sulfasalazine (Azulfidine^®^)

### Solid Dispersions

Solid dispersion indicates the dispersion of one or more drugs in an inert excipient or matrix, in the solid form. It is usually prepared using the melting (fusion), solvent evaporation, co-precipitation, melting–extrusion, or spray drying method (Serajuddin, 2018). Solid dispersions are generally formulated using a hydrophilic polymer and a poor water-soluble drug. In solid dispersions, the physical state of the active pharmaceutical ingredient is notably transformed from the crystalline to amorphous state (Serajuddin, 1999). Solid dispersions of drugs in an amorphous state are rarely eutectic and thus remain metastable and thermodynamically active, leading to their supersaturation in the GI fluid. This leads to a greater concentration gradient and thus increased dynamic force for drug transport across the cellular membrane. Moreover, the dissolution rate and bioavailability of solid dispersions of poorly water-soluble drugs are considerably higher because of the increased surface area and wettability owing to the reduced particle size. This approach can be used for BCS class II drugs that have dissolution rate-limited absorption. The melting method is commonly used for developing scalable quantities of pharmaceutical formulations, but it is not applicable to thermolabile compounds (Serajuddin, 2018). Common pharmaceutical excipients suitable for solid dispersions include cellulosic compounds such as hydroxypropyl cellulose (HPC) or hydroxypropyl-methylcellulose, PEG, polyvinylpyrrolidone, polyvinyl alcohol, and crospovidone (Serajuddin, 1999; Newman, 2015). The bioavailability of orally administered cyclosporine A (CsA), a BCS class II drug, was improved by preparing its solid dispersion formulation using the wet-milling method and HPC hydrophilic polymers. The amorphous solid dispersion of CsA showed significant increase in the C_max_ and AUC to about 5-fold, leading to enhanced therapeutic efficacy in inflammatory disease treatment and organ transplantation (Onoue et al., 2010) A representative list of commercially available oral solid dispersions is shown in Table 7.

**TABLE 7:** Representative examples of solid dispersion formulations.

Technology	Drug molecule	BCS class	Trade name	Formulation	Therapeutic use
Nanocrystal (wet media milling)	Rapamycin	II	Rapamune	Tablets	Immuno-suppressant
	Aprepitant	IV	Emend	Capsules	Antiemetic
	Fenofibrate	II	Tricor	Tablets	Antilipidemic
	Megestrol acetate	II	Megace ES	Oral suspension	Hormonal therapy
High-pressure homogenization	Fenofibrate	II	Triglide	Tablets	Antilipidemic
Melt extrusion	Verapamil HCL	I	Isoptin SRE	Tablets	Antihypertensive
	Nifedipine	II	Adalat SL	Capsules	Antihypertensive
	Troglitazone	II	Rezulin	Tablets	Antidiabetic
Melt adsorption	Nifedipine	II	Afeditab	Tablets	Antihypertensive
Melt granulation (MeltDose^®^ technology)	Fenofibrate	II	Fenoglide	Tablets	Antilipidemic
	Tacrolimus	II	LCP-Tacro	Tablets	Immuno-suppressant
Spray drying	Intelence	IV	Etravirine	Tablets	Antiviral
	Itraconazole	II	Sporanox	Capsules	Antifungal
	Nilvadipine	II	Nivadil	Capsules	Antihypertensive
	Tacrolimus	II	Prograf	Capsules	Immuno-suppressant
Lyophilization	Olanzapine	II	Zyprexa	Tablets	Antipsychotic
	Ondansetron	II	Zofran ODT	Tablets	Antiemetic
	Piroxicam	II	Proxalyoc	Tablets	Anti-inflammatory

### Drug Complexation

Inclusion complex formation with drug molecules is another approach to improve their aqueous solubility; it allows to control the release rates of lipophilic drugs; mask the taste of bitter drugs; and maximize the tolerance of oral drug formulations by minimizing the irritation of the drugs after oral administration (Loftsson and Brewster, 1996). Moreover, it has the added advantage of improving the stability of drugs, especially esters, by shielding chemically labile substances from potentially harsh environmental conditions and reducing their enzymatic degradation, hydrolysis, or oxidation. Generally, cyclodextrins are considered as potential carriers to improve oral delivery of drugs, although other types of complexing agents for instance sodium benzoate, niacin, caffeinate, and salicylate can be used (Loftsson and Duchêne, 2007). Cyclodextrins are chains of cyclic oligomers enclosing 6, 7, and 8 d-glucopyranose structures named alpha, beta, and gamma-cyclodextrins, respectively. Hydroxy-propyl chemical derivatives of β-cyclodextrin have considerable higher aqueous solubility than the native cyclodextrins. β-cyclodextrins are one of the potential complexing agents; their central hydrophobic cavity can be utilized to form inclusion complexes with nonpolar molecules. Thus, they increase the aqueous solubility of drugs that are slightly soluble or water-insoluble to boost their oral bioavailability. At present, more than 85 different oral drug formulations based on complexation are available in the market (Choudhury et al., 2018).

### Ion Pairing (Co-Crystals)

Co-crystals can be described as crystalline solids consisting of two or more molecular and ionic compounds held together by non-covalent forces (Blagden et al., 2007). They might be considered as the crystalline counterpart of solid dispersions. The formation and sustenance of co-crystals in a supersaturated solution can enhance drug absorption and oral bioavailability (Kwei et al., 1995). Orally consumed co-crystals act similar to a single unit and partition into the intestinal membrane as a hydrophobic unit. This approach involves the co-administration of an additional concentration of a counter ion. The ionic compounds dissociate when diluted after administration in the GI medium. In principle, this strategy is simple and eliminates the need for chemical modification or prodrug design. Ion pairs need to have desired characteristics such as high lipophilicity, sufficient aqueous solubility, biocompatibility, and physiological stability. The most commonly used counter ions are phthalic acid, succinic acid, and benzoic acid. However, these counter ions used for ion pairing can compete with endogenous compounds such as sialic acid, bile acids, and phosphoglycerides (Varshosaz et al., 2018). In addition, most counter ions are not safe, which could cause membrane irritation and toxicity, particularly at higher doses. Ion pairing by using naphthoic acid as a counter ion has been used to deliver highly polar antiviral drugs (Miller et al., 2010). A study showed that itraconazole cocrystals with succinate, maleate, and tartrate behaved in a identical manner to the amorphous form, however its solubility improved from fourth to twentieth fold in comparison to that of the crystalline form of the drug (Remenar et al., 2003).

#### Absorption Enhancers

Various absorption enhancers are known to increase drug permeability in the intestine, especially for BCS class III drugs. Compounds such as surfactants, cholesterol, glycerides, salicylates, bile salts,, and chelating agents are used as absorption enhancers (Aungst, 2012). Most absorption enhancers increase the transport of hydrophilic drugs by altering their paracellular permeability (LeCluyse and Sutton, 1997). However, some absorption enhancers might cause mucosal damage and systemic toxicity. Ethylene diamine tetraacetic acid (EDTA) is commonly used as a paracellular permeation enhancer to deplete calcium and magnesium in the tight junctions (Lemmer and Hamman, 2013). However, strategies involving the modulation of the permeability of tight junctions have safety concerns. Once tight junctions are opened, not only drugs but also other toxic molecules can be transported across the intestinal membrane. Conversely, transcellular promoters can increase the oral absorption of drugs primarily by fluidizing, solubilizing, or reorganizing the intracellular phospholipids, causing the disruption of membrane integrity. Examples of such enhancers include tartaric acid, sodium salicylate, sodium caprate, and sodium caprylate.

#### Metabolism and Efflux Pump Inhibitors

The co-administration of metabolism and efflux pump inhibitors has been shown to overcome multidrug resistance and increase the oral bioavailability of drugs that are substrates of efflux transporters such as P-glycoprotein, MRP2, and BCRP (Pang, 2003). Recently, many excipients have been shown to modulate the function of efflux transporters, such as polyethoxylated castor oil (Cremophor EL), polysorbates (Tweens), poloxamers (pluronic P85), tocopherol polyethylene glycol 1,000 succinate (TPGS 1000), and polyethylene glycol (PEG) (Murakami and Takano, 2008). Recent studies on oral delivery have been focusing on exploring the ability of pharmaceutical polymers to inhibit the efflux pump activity (Werle, 2008; Dahlgren and Lennernäs, 2019). Although the exact mechanism of P-gp inhibition is not yet known, polymeric excipients might act via one or more mechanisms that include competing with the substrate-binding site on the efflux transporter, altering the membrane lipid fluidity, protecting the drug while by-passing the efflux transporter, inhibiting the efflux pump ATPase activity, or directly acting on the P-gp protein on the mucosal surface (Takano et al., 2006). Examples of P-gp substrates include cyclosporine A and vancomycin hydrochloride formulated with TPEGS. These studies indicate that the improved oral bioavailability of drugs administered with TPEGS might, in part, be attributed to the inhibition of P-gp pump (Dintaman and Silverman, 1999). In one study, a formulation containing paclitaxel and TPEGS in 1:2 ratio led to 6-fold improvement in the oral bioavailability of paclitaxel to approximately 32% in rats (Varma and Panchagnula, 2005). Mucoadhesive polymers such as dextran, chitosan, polycarbophil, and polyacrylic acid have been shown to affect the activity of intestinal protease enzymes, mainly trypsin, chymotrypsin, and carboxypeptidases, which might be useful for the oral delivery of metabolically labile drugs as well as increase their residence time. The co-administration of grapefruit juice and ketoconazole was shown to reduce the pre-systemic metabolism by CYP3A4 present in the intestinal enterocytes (Dresser et al., 2000). Similarly, the administration of erythromycin was found to increase the oral bioavailability of cyclosporine by selectively inhibiting hepatic CYP3A4 metabolism. Even though this approach seemed to be effective, the co-administration of metabolism inhibitors and dietary components such as grapefruit juice might not likely be approved by the FDA and other regulatory agencies for routine clinical practice. Initially, drugs were not particularly developed to inhibit P-gp activity; basically, they had other therapeutical properties, as well as a low affinity for transporters. For instance, the first-generation P-gp inhibitors such as verapamil (Nobili et al., 2006). However, most of these drugs were less specific and had more adverse effects. Second generation P-gp inhibitors such as biricodar (VX-710) are potent and selective, but have adverse pharmacokinetic interactions (Leonard et al., 2002). Several third generation P-gp inhibitors such as zosuquidar (LY3359797) and tariquidar (XR9576) have been developed and ongoing studies being explored in clinical studies (Planting et al., 2005; Wang et al., 2014).

#### Lipid-Based Formulations

Lipid-based formulations have been used for the oral administration of drugs that are poorly soluble in water, such as BCS classes II and IV drugs. Such formulations represent 3% of the total drug products available in the market (Hauss, 2013). Depending on their composition, size, and chemical characteristics, lipid-based systems can be further classified into lipid solutions, lipid suspensions, emulsions, multiple emulsions, micro- and nanoemulsions, self-emulsifying and self-micro-emulsifying systems, solid lipid nanoparticles, solid lipid dispersions, niosomes, and liposomes. Lipid-based formulations are an attractive approach for oral application owing to their inherent biocompatibility, particle size versatility, scaling-up ability, and cost-effectiveness (Hauss, 2007). A representative list of lipid-based formulations marketed for oral administration is shown in Table 8. Most of these formulations can be administered as liquid-filled hard capsules or tablets as well as oral liquids in the form of solutions or suspensions. Moreover, these dosage forms can be utilized for sustained- or immediate-release formulations. A lipid-based carrier is effective for the oral delivery of small hydrophobic molecules via several mechanisms. One of the main mechanisms is to enhance the dissolution rate and solubility in the GI tract. The digestion of lipids is started in the stomach by gastric lipases. Shear forces in the digestive tract and stomach emptying assist in the emulsification of the drug before emptying into the duodenum. Secretion of pancreatic enzyme lipase together with its co-factor co-lipase facilitates the breakdown of intake glycerides to diglycerides, monoglycerides, and fatty acids. The existence of fatty diets in the intestine also stimulates the gallbladder biliary secretions of bile salt, cholesterol, and phospholipids. Due to the presence of bile salt, the yields of lipid digestion are consequently assembled into a colloidal structure, including vesicles, mixed micelles, and micellar carrier. These carriers increase the solubilization of the drugs in the intestine. Furthermore, the nature and composition of the formulation (such as lipids, surfactants, co-solvents, and complexation agents) as well as bile salts and phospholipids contribute to the enhanced absorption (Hauss, 2007; Savla et al., 2017). Thus, lipid-based formulations maintain a higher drug concentration gradient for facilitating the diffusion of drugs across the unstirred aqueous layer and then to the mucosal membrane. Interestingly, the co-administration of a fatty food has also been shown to provide similar advantages to improve drug dissolution and bioavailability (McClements and Xiao, 2014). This also explains why most lipid-based formulations have reduced food effects compared to conventional oral formulations (Fatouros et al., 2007). Poorly water-soluble drugs administered using lipid-based systems can be protected against enzymatic degradation. Interestingly, lipid-based carriers such as liposomes have been shown to be absorbed in an intact state by pinocytosis across the epithelial membrane and occasionally through the lymphatic system (Lee, 2020). Lymphatic transport of lipophilic drugs (e.g., cyclosporine) occurs mainly through the mesenteric lymph and can thus avoid the hepatic first-pass metabolism (O’Driscoll, 2002). In addition, pancreatic lipase digestion results in the breakdown of triglycerides into monoacylglyceride and free fatty acid molecules that can interact with fatty acid transporters present on the apical membrane and mediate drug absorption. While the common forms of lipids such as cholesterol, phospholipid, and tocopherol are absorbed via the chylomicron pathway, the majority of lipids and lipid digestion products are absorbed via fatty acid transporters (Porter et al., 2007). Recently, more evidence suggests that lipid-based formulations inhibit the efflux transporter P-gp (Sachs-Barrable et al., 2007). This might be beneficial for BCS class IV drugs that are substrates of the P-gp transporter pump. In addition to improving the oral bioavailability of BCS class II/IV drugs, several lipid derivatives such as stearic acid, oleic acid, hydrogenated castor oil, and glyceryl trimyristate have been developed to sustain the release of water-soluble drugs (Jannin et al., 2008). The sustained-release formulations prepared as a semi-solid lipid matrix filled in hard gelatin capsules seem to prolong the absorption of drugs even under a fed state, which could be attributed to the delay in the gastric emptying effect. Although lipid-based delivery systems have the potential as drug carriers, obtaining the overall consistency, including in physicochemical properties, drug encapsulation, drug-release kinetics, and particle size, is difficult, especially with liposomes and solid lipid nanoparticles (Phan et al., 2014). Furthermore, the ability of lipids to incorporate drugs differs in regard to their crystallization and polymorphism properties, which usually results in undesirable interactions and inconsistency (Parve et al., 2014). In addition, the availability and range of lipid-based excipients is limited (Dahan and Hoffman, 2008). Another major disadvantage of lipid-based systems is the physical and/or chemical instability issues, especially during long-term storage and handling.

**TABLE 8:** Representative examples of marketed oral lipid-based formulations.

Lipid-based formulation	Drug molecule	BCS class	Trade name	Therapeutic use
Solutions	Dutasteride	II	Avodart (GlaxoSmithKline)	Hyperplasia of prostate
	Efavirenz	II	Sustiva (Bristol–Myers Squibb)	Antiviral
Suspensions	Clofazimine	II	Lamprene (Novartis)	Leprosy
	Isotretinoin	II	Accutane (Roche)	Acne vulgaris
	Progesterone	II	Prometrium (AbbVie)	Hormone therapy
	Bexarotene	II	Targretin (Ligand Pharmaceuticals)	Antineoplastic
	Tocopherol nicotinate	–	Juvela (Eisai)	Hypertension, hyperlipidemia
	Valproic acid	I	Convulex (Pharmacia)	Antiepileptic
	Ciprofloxacin	IV	Cipro (Bayer)	Antibiotic
SEDDS	Cyclosporine A	II	Sandimmune (Novartis)	Immunosuppressant
	Ritonavir	IV	Norvir (AbbVie)	Antiviral
	Saquinavir	IV	Fortovase (Roche)	Antiviral
	Lopinavir	IV	Kaletra (AbbVie)	Antiviral
SMEDDS	Cyclosporine A	II	Neoral (Novartis)	Immunosuppressant

### Polymeric Micellar Carriers

Drug solubility can be enhanced and drug precipitation after exposure to the GI environment can be avoided by incorporating poorly soluble compounds in surface-active agents, known as copolymers. Micellar systems occur in dynamic equilibrium in three systems in a surfactant solution: monomeric surfactant, micellar aggregates, and surfactants adsorbed as a film at the interface. Micellar carriers are formed when the concentration of surfactant is above the critical micellar concentration (CMC) (Ribeiro et al., 2018). Amphiphilic copolymers comprising of hydrophobic and hydrophilic blocks freely associated into micelles when dissolved in an aqueous environment. The hydrophobic domains of the copolymers form the core and the hydrophilic tails form the external shell of the micelles. The lipophilic core serves as a container for loading lipophilic drugs, whereas the corona stabilizes the interface between the hydrophobic drug and aqueous medium. Micellar carriers can be utilized to increase the solubility of lipophilic drugs by incorporating them in the micellar core (Gaucher et al., 2005). Recently, amphiphilic block copolymers have been developed as solubility enhancers (Simões et al., 2014). Poloxamers surfactant series is one of the most commonly used block copolymers in drug delivery. The CMC of these copolymers range from 10^–5^ to 10^–8^ M, whereas that of standard low molecular weight surfactants range from 10^–3^ to 10^–4^ M (Kwon and Okano, 1996). As micelles have the CMC of 10^–6^ M, their ability to withstand dilution than is greater than that of surfactants with low molecular weight. In one study, pH-sensitive micellar carriers were used to enhance the oral bioavailability of a hypercholesterolemia drug, fenofibrate by 15% compared to that of its coarse suspension. The study showed that the micellar composition was important in determining the desired drug release profile (Sant et al., 2005). Moreover, these micellar systems can be chemically modified through the conjugation of antibodies on their side chains for improved target specificity. It is worth noting that antibody-conjugated micelles may undergo rapid clearance from blood circulation due to their accumulation in the liver, mainly in the absence of adequate target antigens (Musacchio and Torchilin, 2019).

#### Polymeric Nanocarriers

The exponential development of nanotechnology has allowed the development of new oral drug delivery systems. Numerous natural or synthetic based polymers have been utilized to prepare oral drug delivery systems. Some natural polymers commonly used include dextran, chitosan, gelatin, and alginate, and the synthetic based polymers used as oral drug delivery carriers include polylactide-coglycolide (PLGA), polylactide (PLA), polycaprolactone (PCL), polyglycolide, polycyanoacrylate, and polyaziridine (Ritika et al., 2012). The nanotechnology approach involves the formulation of drugs by using particles that are in the nanometer size range of 10–1,000 nm. Nanocarriers can be prepared using many methods that can be divided into two groups.

Top-down techniques: They are based on the reduction of the particle size of relatively large polymers into small particles; they involve processes that apply high shear, ultrasonication, cavitations, homogenization, microfluidization, spray drying, or milling (Pinto Reis et al., 2006).

Bottom-up techniques: They are based on the growth of particles formed from individual particles; they are mainly known as phase separation methods. Examples include spray-freezing liquid, coordinated crystallization during freeze-drying, and pharmaceutical technologies that are based on supercritical fluid (de Waard et al., 2011).

**FIGURE 2:**
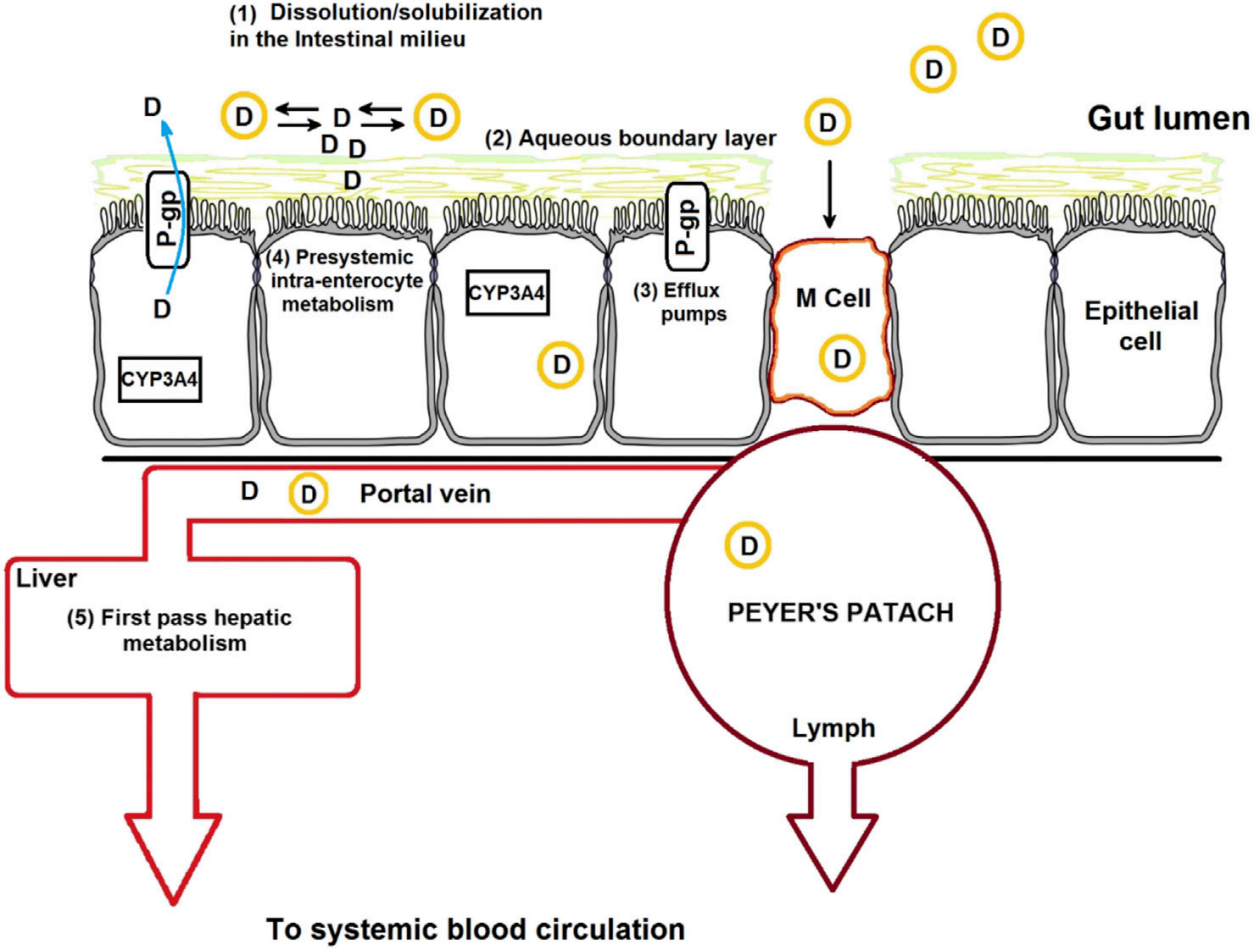
Schematic representation of the drug absorption barriers and mechanisms of nanoparticle transport across the intestinal epithelium, which include transcellular transport, receptor-mediated transport, and M-cell-mediated transport.

Reducing the particle sizes to the nanometer scale results in larger effective surface area, eventually enhancing the dissolution rate and solubility of drugs (Mei et al., 2013). Examples of nanocarriers used for the oral drug administration are shown in Table 9. Polymeric nanocarriers can be used to deliver insoluble drugs, target the drugs to specific regions of the GI tract, minimize food effect on drug absorption, facilitate transcytosis of drugs across the mucosal membrane, and permit receptor-mediated intracellular drug delivery (Mei et al., 2013; Ottenbrite and Kim, 2019). Another attribute that makes micro/nanocarriers efficient oral drug carriers is that they can carry a wide array of agents for diagnosis and therapy, ranging from small molecules to peptides, proteins, and nucleic acids, and releasing them in a controlled manner. Thus, polymeric nanocarrier-based drug delivery can enhance the specificity, tolerability, and efficacy of therapeutic agents. Examples of patented nano-formulations employed for the oral delivery of drugs are shown in Table 10. A continues knowledge of the mechanisms implicated in micro/nanocarriers uptake is required for the design of novel nanocarriers and new-targeted systems for the oral route. A schematic representation of different barriers to oral drug delivery and micro/nanocarriers uptake mechanisms in the intestine are shown in (Figure 2). In general, particles or drugs reach the blood circulation when they are absorbed via the enterocytes, whereas intact particles are delivered to the lymphoid tissue after being transcytosed via M cell uptake which occurs in the Payer’s patch (Brayden et al., 2005). Nanoparticle uptake by enterocytes or M cells and stability in the GI tract depend mainly on the particle size, surface characteristics, molecular weight, and chemical composition (Kulkarni and Feng, 2013). For oral drug delivery, particle size plays an important role because it influences particle adhesion and interaction with the mucosal membrane and the drug-release kinetics (Kulkarni and Feng, 2013; Alqahtani, 2017). Particles having sizes below 50, 100–500 nm, and below 5 μm pass through the GI barriers via paracellular channels, endocytosis by enterocytes, and cellular uptake by M cells of the Peyer’s patch, respectively (Desai et al., 1996). Several studies have indicated that the uptake of particles with size diameters of 100 nm is higher than that of larger particles in the rat GI mucosa (Desai et al., 1996; Janer et al., 2014). Biodegradable polymers used in drug delivery have many advantages over non-biodegradable polymers; the former are safe and undergo complete degradation after drug release. For example, PLGA nanoparticles degrade into lactic acid and glycolic acid via hydrolysis, polycyanoacrylate nanoparticles degrade into cyanoacetate and formaldehyde, and protein nanoparticles are enzymatically degraded to basic amino acids and peptides (Ratnaparkhi and Gupta Jyoti, 2013). Polymers for oral delivery of drugs often have an upper limit on the concentration of polymers that is nontoxic (Islam et al., 2019). For sustained release applications of potent drugs, the precise concentration of drug desired should be calculated and the formulation optimized accordingly by tuning the polymeric constituents or formulation process. Bioerosion or swelling of polymers results in the diffusion of drugs from the nanoparticles in tunable release profile. Polymers can be modified to exhibit the preferred release profile through cross-linking or chemical conjugation of the encapsulated drug (Alqahtani et al., 2019). Polymers can also be combined with hydrogels or scaffolds to further fine-tune the desired release profile (Liu, 2018). Biopolymers such as protein-based nanoparticles have been used owing to their desirable features, including generally recognized as safe (GRAS) status and biodegradability and correspond to amino acids. Some protein-based polymers that have been investigated for oral delivery include whey proteins, casein, gelatin, soy proteins, zein, and wheat proteins (Shukla and Cheryan, 2001; Dong et al., 2004). They can be used as drug delivery systems and in the food industry (Hurtado-Lopez and Murdan, 2005; Liu et al., 2005; Parris et al., 2005; Wang et al., 2005; Lai and Guo, 2011; Chen et al., 2014). Furthermore, the amphiphilic characteristic of proteins allows them to interact equally well with both the drug and intestinal mucosa (Islam et al., 2019). Therefore, nanoparticles obtained from natural proteins have an enhanced tendency for biological interaction and facilitate surface modification for the attachment of numerous drugs and target specific areas by using ligands. Protein-based nanoparticles have been synthesized using both water-soluble and non-soluble proteins (Perumal and Podaralla, 2019). Human serum albumin and bovine albumin are examples of water-soluble carriers, whereas zein and gliadin are lipophilic carriers. However, the use of protein polymers for oral drug delivery has been less studied, especially with regard to the understanding of enzymatic stability, drug-release kinetics, and absorption mechanism. The challenges relate to nano-formulations yield, polydispersity, sonochemistry and throughput, are still the biggest obstacle against the widespread application of these systems, this prevents them from being scaled up to commercial levels and from entering the mass production for pharmaceutical industry (Pathak and Thassu, 2016).

**TABLE 9:** List of nanocarrier applications in oral drug delivery.

Nano-system	Composition	Drug molecule size (nm)	Size (nm)	Cell line/animal model	Disease or targeted organs	References
Dendrimers	G3.5 PAMAM	SN38	–	Caco-2 cells and HT-29/female CD-1 mice	Colorectal cancer metastases	(Goldberg et al., 2011)
	Ethylene diamine and Methyl acrylate	SN38 camptothecin	13	CD-1 mice	Oral chemotherapy of hepatic colorectal cancer metastases	(Sadekar et al., 2013)
	PAMAM	Short hairpin RNA	107–315	Tca8113 cells/BALB/c nude mice	Oral cancer therapy	(Liu et al., 2011)
Mic(Sadekar et al., 2013)e lles	Polyethylene oxide–polypropylene oxide–polyethylene oxide (PEO–PPO–PEO)	Paclitaxel	180	Female C57BL/6J mice	Oral cancer therapy	(Yoncheva et al., 2012)
	*N*-octyl-*O*-sulfate chitosan (NOSC)	Paclitaxel		Caco-2/SD rats	Improved oral bioavailability	(Mo et al., 2011)
	Bovine β-casein	Celecoxib, Paclitaxel	20	Human N-87 gastric cancer cells	Rheumatoid arthritis, osteoarthritis, and gastric carcinoma	(Bachar et al., 2012; Shapira et al., 2012)
	Tocopherol succinate glycol chitosan conjugates	Ketoconazole	101	Caco-2 cell monolayer	Improved oral bioavailability	(Duhem et al., 2012)
Mixed Micelles	Pluronic copolymers and LHR conjugate	Paclitaxel	140	MCF-7 cells	Oral anticancer delivery system	(Dahmani et al., 2012)
Vesicles	PLA-P85-PLA	Insulin	178	OVCAR-3 cells/diabetic mice	Oral insulin delivery	(Xiong et al., 2013)
Liposomes	Lecithins	Curcumin	263	Sprague–Dawley (SD) rats	Improved oral bioavailability	(Takahashi et al., 2009)
SLN	lyceryl monostearate (GMS)	Vinpocetine	70–200	Male Wistar rats	Improved oral bioavailability	(Luo et al., 2006)
Polymeric microspheres	Chitosan and alginate	Insulin	5–7 μm	Male SD rats	Diabetes mellitus	(Zhang et al., 2011)
Polymeric nanoparticles	PLGA	Cyclosporine	143 nm	Male SD rats	Improved oral bioavailability	(Italia et al., 2007)
	Silica	Resveratrol	90 nm	Caco-2 cell monolayer	Enhanced the solubility, permeability and anti-inflammatory activity of resveratrol encapsulated in NPs	(Juere et al., 2017)
Polymeric microspheres	Chitosan and alginate	Insulin	5–7 μm	Male SD rats	Diabetes mellitus	(Zhang et al., 2011)
Polymeric nanoparticles	PLGA	Cyclosporine	143 nm	Male SD rats	Improved oral bioavailability	(Italia et al., 2007)
	Silica	Resveratrol	90 nm	Caco-2 cell monolayer	Enhanced the solubility, permeability and anti-inflammatory activity of resveratrol encapsulated in NPs	(Juere et al., 2017)
Multifunctional polymeric nanoparticles	Galactose-modified trimethyl chitosan–cysteine conjugates with various galactose grafting densities	shRNA and siRNA	130–160 nm	Caco-2 cells/tumor-bearing mice	Targeted treatment of hepatoma	(Han et al., 2014)
	Mannose-modified trimethyl chitosan-cysteine (MTC) conjugate	Tumor necrosis factor-α (TNF-α) siRNA	152.9 nm	Caco-2 cells, RAW 264.7 (monocyte/macrophage-like cells)/ acute hepatic injury induced mice	Treatment of systemic inflammatory conditions	(He et al., 2013)
	Lectin-conjugated PLGA-NPs	Betamethasone	475 nm	TNBS-induced induced colitis mice	Treatments of ulcerative colitis and inflammatory bowel disease	(Moulari et al., 2014)

**TABLE 10:** List of patented formulations related to nanoparticles for oral drug delivery.

Patent number	Assignee	Invention	References
WO2008073558A2	Johns Hopkins University, USA	The invention provided new orally bioavailable smart NPs for delivery of poorly soluble drugs, showing improved pharmacokinetics and bioavailability	(Maitra et al., 2014)
WO2015057751A1	NanoSphere Health Sciences Inc., USA	Investigation disclosed the composition and development method for nutraceuticals encapsulated within phospholipids-based NPs by emulsification Method	(Kaufman, 2018)
US20120003306A1	NanoMega Medical Co., USA	The report disclosed a protein/peptide delivery system composed of chitosan and poly-γ-glutamic acid (γ-PGA). The NPs were suggested to enhance the epithelial permeability and thus are efficient for oral drug delivery	(Sung et al., 2012)
WO2004098564A2	University of Illinois, USA	Reported the development of biodegradable NPs containing streptomycin with high loading efficiency of 50% or higher for tuberculosis treatment. The NPs also can contain other aminoglycosides drugs, which are a known substrate for the multidrug efflux P-glycoprotein (Pgp)	(Popescu and Onyuksel, 2004)
US7674767B2	Samyang Biopharmaceuticals Co., Korea	The invention described the compositions and preparation of orally administrable NPs containing complexes of water-soluble drugs and counter-ion substances. The NPs enhanced drug entrapping and resistance against lipases, thereby increased drug absorption	(Pai et al., 2010)
WO2015023797A9	Northwestern University, USA	The patent disclosed the development and evaluation of drug loaded nanostructures comprising an inorganic core and a lipid layer shell. The NPs showed the potential in the treatment of cancer, vascular disease and infectious disease	(Thaxton et al., 2020)
WO2014197640A1	South Dakota State University, USA	Disclosed the composition and preparation method of core-shell NPs. These NPs comprising food grade proteins along with therapeutic agent suitable for pediatrics	(Perumal and Alqahtani, 2015)
WO2007042572A1	Advanced *In Vitro* cell Technologies S.A., Spain	The invention described NPs comprising chitosan and heparin prepared by ionic gelation method. The NPs were stable in gastrointestinal fluids and presented an excellent *in vivo* effectiveness and bioavailability	(Pena et al., 2008)
CN102120781B	China Pharmaceutical University, China	The invention related to the preparation of oral insulin NPs. The NPs mainly contained N-amino acid chitosan as a carrier and insulin for the treatment of diabetes. The NPs were stable after oral administration with a better effect of reducing blood sugar *in vivo*	(Zhang et al., 2013)
US10420731B1	King Saud University, Saudi Arabia	The invention disclosed the synthesis and preparation method of lignin NPs crosslinked and stabilized by citric acid for oral administration. The NPs improved the oral bioavailability of curcumin by increasing curcumin solubility, stability, sustained its release, enhanced intestinal permeability, and inhibition of P-gp mediated efflux	(Alqahtani et al., 2019)
WO2011034394A2	JW Pharmaceutical Co., Korea	The invention reported the preparation of oxaliplatin-loaded NPs using supercritical fluid gas technology for oral chemotherapy	(Lee et al., 2016)
WO2010015688A1	BioAlliance Pharma Co, USA	The patent disclosed the composition and preparation method of chemotherapeutic formulation containing polymer and cyclic oligosaccharide capable of complexing and delivering anticancer drugs for effective cancer treatments	(Bonnafous et al., 2011)

## Pediatric Oral Drug Delivery

Pediatric patients represent 23% of the total US population and consist of newborns babes, infants, children, and adolescents (Madill, 2017). The upper age limit used to distinguish the pediatric population differs between countries; usually, adolescents are considered up to the age of 18 or 21 years. Different age groups have different physiological and pharmacokinetic considerations, as well as ability to handle formulations. Although efforts have been made to develop and design new pediatric formulations, the development of an acceptable pediatric formulation remains a challenging task. Furthermore, the development of pediatric formulations is hindered by the absence of market share or economic stimulus, since the majority of the patients is comprised of adults; dilemma in developing formulations that are adequately taste-masked; methodology and ethical requirements for clinical trials in children; and high costs associated with development research, manufacturing, and storage (Strickley, 2019). Many physiological and maturational changes occur in growing children. These age-related changes impact the absorption, disposition, and metabolism of drugs. For example, the pH of the GI tract is different between adults and children (Lam et al., 2013). Similarly, differences also exist in gastric residence time, gastric emptying time, intestinal transit time, P-gp expression in the GI tract, and bacterial population composition (Strolin Benedetti and Baltes, 2003). In addition, differences exist in the total body water content, enzymatic activity, and blood flow as well as in fat content. From the formulation prospective, many pharmaceutically active ingredients have poor water solubility, stability, or an unpleasant taste, thereby rendering pediatric drug development a challenging task. Children cannot swallow large tablets and capsules and are unable to accept formulations that taste bitter or bad. In addition to children, many geriatric and ill adults, such as patients in surgeries or recovering from comas, also unable to swallow tablets and capsules, thereby expanding the need for pediatric formulation. Clinically desirable pediatric dosage forms involve a solid dosage form or an orally dissolvable formulation that is tasteless; formulated with safe and minimal additives; and in a appropriate dosage form that is stable even after exposure to heat and humidity. Liquid pediatric dosage forms have limited storage stability, palatability, and handling (Standing and Tuleu, 2005). Conversely, solid formulations such as powders or granules are devoid of these issues. In 1937, sulfanilamide was formulated into a solution by using the diethylene glycol (DEG) as a solvent, which resulted in the death of around 100 children. Diethylene glycol is still used in developing countries owing to the lack of pediatric formulations, causing pediatric deaths (Nahata, 1999). Hence, pediatric drug formulations have attracted considerable attention from private and government institutes. Excipients are the inactive ingredients in drug formulations and should be safe for human use. However, some additives that are safe in adults might not be suitable for pediatrics, especially in infants and newborns babes owing to their physiological features and age-related maturation of tissue functions (Nunn, 2003). Further, as child friendly formulations are inadequate, incompliance to the prescribed regimen of medication is noted in children, resulting in the lack of therapeutic effect (Standing et al., 2005). This is especially critical in the case of cancer or infectious diseases in the pediatric population. Hence, to date, parents and nurses continue to use extemporaneous compounding to help children take the current adult formulations.

In 1997, the FDA implemented pediatric regulations and legislation in the US. Furthermore, draft guidance on pediatric clinical studies have been published by the American Academy of Pediatrics and FDA (Food and Drug Administration, 1998) (Shaddy and Denne, 2010). In addition, the World Health Organization (WHO) published a position report for desired pediatric dosage forms and integrated it in a Model Essential Medicines List for Children (World Health Organization, 2010). This report stated a policy on oral pharmaceuticals suitable to children, especially in the developing countries, and specified the need for solid oral dosage forms for easy transport in pediatrics, since they do not require refrigeration, are easy to reconstitute with tap water, and can be given at the right dose, such as orally disintegrating dosage forms, multi-particulate formulations in the form of powders/sprinkles, mini-tablets, and chewable tablets. However, formulations for pediatrics are not commercially available in the developing world owing to the difficulties in the development and storage of such formulations (Sosnik et al., 2012). Although most pediatric formulations of BCS classes 1 and 3 drugs are in the liquid form, many drugs, including those for chemotherapy or HIV, belong to BCS classes II and IV, which are poorly soluble in water and bitter in taste, and thus pose challenges in oral drug development (Milne and Davis, 2014).

Drug solubility and taste can be altered by chemically modifying, masking by encapsulation, or adding sweeteners to the drugs. Drug absorption is affected by the choice of polymers or excipients. In addition to taste masking, polymeric encapsulation can improve the drug solubility and stability and allow controlled drug release and absorption (Weuts et al., 2011). These polymers can either be natural or synthetic or act as a barrier to control drug release and prevent the degradation of drugs after oral administration or during storage. A list of marketed oral pediatric formulations is shown in Table 11.

**TABLE 11:** Representative list of marketed pediatric formulations.

Oral pediatric formulations	Technology and advantages	Examples
Disintegrating tablets (DTs)	DTs are designed to disintegrate in the oral cavity usually in less than a minute. Different than regular tablets that need to be swallowed essentially, DTs reduce the need of tablet swallowing and therefore appropriate for older children. They can be manufactured using different techniques, including freeze-drying process, compaction process and molding candy process	Prevacid^®^ (lansoprazole), Zofran^®^ (ondansetron), and Clarinex^®^ RediTabs^®^ (desloratadine)
Disintegrating films or strips (DFs)	DFs is a strip that disintegrates quickly in the tongue without requiring chewing or swallowing. The casting method and hot-melt extrusion method are used to prepare ODFs	PocketPaks^®^ (Listerine), Triaminic Thin Strips^®^ (Phenylephrine HCl), Theraflu^®^ (Diphenhydramine HCl), Setofilm^®^ (Ondansetron)
Mini-tablets	Mini-tablet is an alternative to address swallowing difficulty in pediatric patients. It has a less than 3 mm diameter. As a substitute of regular tablets, mini-tablets can be easily swallowed by patients less than 10 years old. Its advantage is the ease of manufacturing by using a regular tablet compression machine	Lamisil^®^ (terbinafine), Orfiril long^®^ (Valproic Acid sodium)
Multi-particulates (constituted to a suspension or as sprinkles in apple sauce or yogurt)	Small particles (e.g., granules or pellets) can be mixed with food matrices. Multi-particulates are formulated using pearl milling, phase separation, or high-pressure homogenization	Medikinet^®^ (methylphenidate), Artequin Pediatric^®^ (mefloquine), Lopimune Sprinkles^®^ (lopinavir/ritonavir)

**FIGURE 3:**
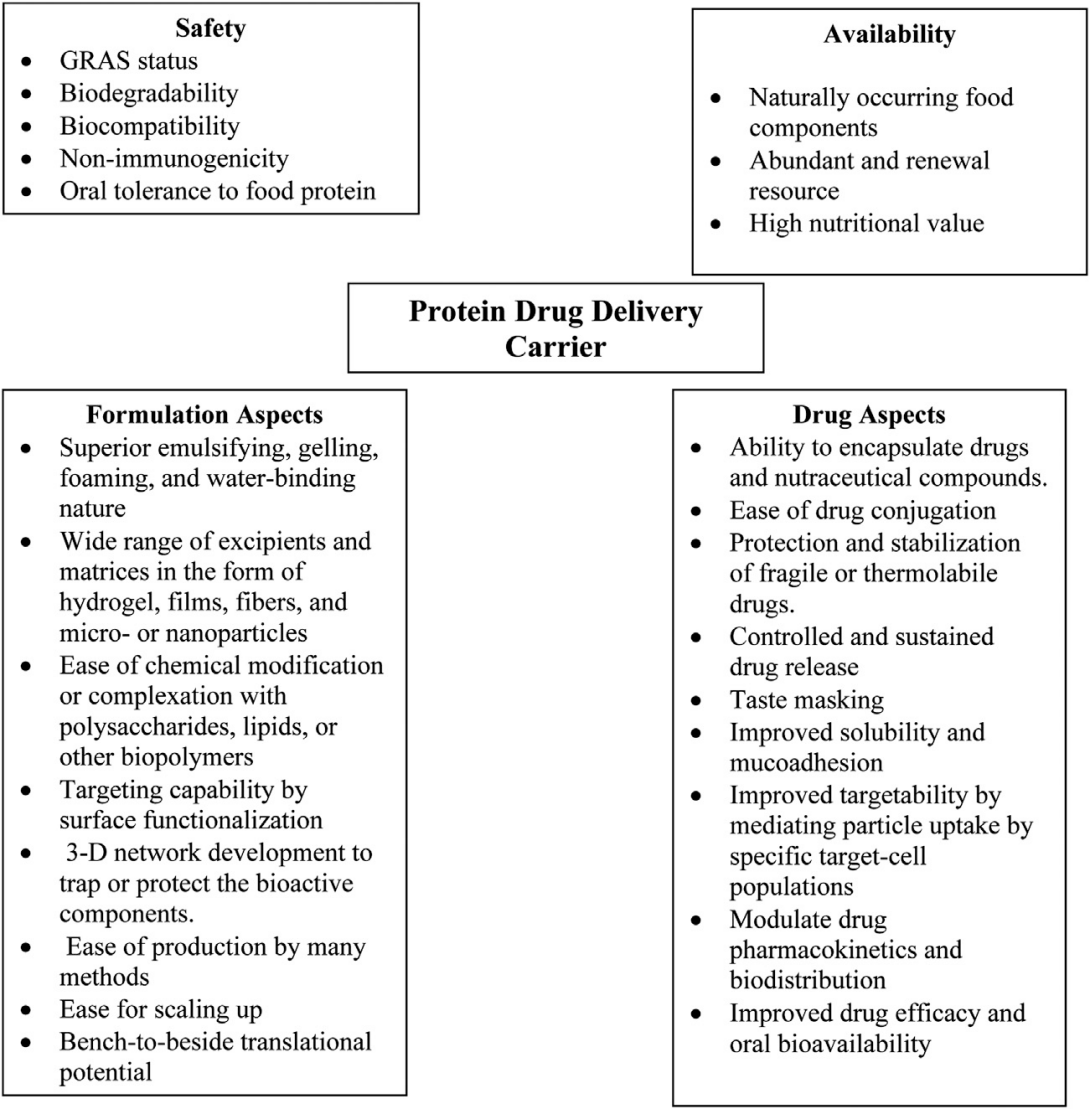
Advantages of proteins as drug delivery carriers.

Oral bioavailability of solid dispersible forms of drugs for pediatric use can be improved by using nanoparticle technologies (Das and Chaudhury, 2011). Novel nanoparticle formulations for treating child cancers, infections, and asthma have shown superior advantages (Yellepeddi et al., 2019). A recent study showed the efficacy of dexamethasone-loaded polymeric nanoparticles to potentially treat childhood leukemia. The nanoparticles made of the copolymer of PEG and PCL to deliver dexamethasone effectively induced cell death and improved survival in pediatric population, even at a low dose of the drug (Krishnan et al., 2012). Food-grade proteins are a versatile class of biopolymers and are generally recognized as safe (GRAS) (Abd El-Salam and El-Shibiny, 2012; Alqahtani et al., 2017). Their main benefits as drug delivery carriers, especially for oral drug delivery, include edibility, safety, and biodegradability. The advantages of using proteins as drug delivery carriers are listed in (Figure 3). Moreover, milk proteins such as caseins possess many favorable characteristics suitable for the development of polymeric nanocarriers, such as amphiphilicity, biodegradability and good biocompatibility, particularly for pediatrics (George et al., 2019). However, studies on the use of food-based proteins along with other GRAS excipients to develop nanocarriers for pediatric drug delivery are far away from the clinical translation.

## Conclusions and Future Perspectives

Oral drug delivery is one of the most common route of drug administration in both adult and pediatric patients. Conventional oral formulations can raise issues and complications that could be addressed by advanced formulation strategies. One such strategy is the use of nanocarriers that could improve drug solubility, permeability and bioavailability. Better understanding of the effects of common diet and inter-patient variation in drug absorption is needed. In vast preclinical studies, the transition from the fed to the fasted state is overlooked, which can affect the mechanism and rate by which drug compounds are absorbed. One critical aspect that still deserves more consideration in the future is the establishment of a reliable *in vitro*-*in vivo* correlation models to predict better *in vivo* performance and to generate data that offer cost benefit over existing formulations. This will help accelerate the transition of more realistic and more relevant formulations from laboratory to commercial production scale. Additionally, the target population of patients must be taken into account when designing new formulations. Future research can be directed toward the development of better pediatric formulations by using nanoparticle technologies that are currently used for developing drug formulations for adults. Prospect studies on nanocarriers technology to develop oral formulations need to consider the use of safe and effective excipients. As the landscape of delivery technologies changes, formulation development and excipient screening will continue to evolve consequently. It is expected that the overall time for formulation development will be shorter than the current existing one to bring a lead compound from drug discovery to clinical trials. However, there are numerous obstacles that pharmaceutical researchers will have to face to accomplish with better and more effective oral formulations that can provide better therapy.
